# Advances in refunctionalization of erythrocyte-based nanomedicine for enhancing cancer-targeted drug delivery

**DOI:** 10.7150/thno.36510

**Published:** 2019-09-21

**Authors:** Da Sun, Jia Chen, Yuan Wang, Hao Ji, Renyi Peng, Libo Jin, Wei Wu

**Affiliations:** 1 Institute of Life Sciences, Wenzhou University, Wenzhou, 325035, China.; 2 Biomedical Collaborative Innovation Center of Zhejiang Province & Engineering Laboratory of Zhejiang Province for Pharmaceutical Development of Growth Factors, Biomedical Collaborative Innovation Center of Wenzhou, Wenzhou, Zhejiang, 325035, China.; 3 Sichuan Provincial Center for Mental Health, Sichuan Academy of Medical Sciences & Sichuan Provincial People's Hospital, Chengdu, 610072, China.; 4 Chongqing Business Vocational College, Chongqing, 401331, China.; 5 Key Laboratory for Biorheological Science and Technology of Ministry of Education, State and Local Joint Engineering Laboratory for Vascular Implants, Bioengineering College of Chongqing University, Chongqing, 400030, China.

**Keywords:** erythrocytes, nanomedicine, refunctionalization, cancer

## Abstract

Cancer remains a daunting and cureless disease, which is responsible for one-sixth of human deaths worldwide. These mortality rates have been expected to rise in the future due to the side effects of conventional treatments (chemotherapy, radiotherapy, and surgery), which can be addressed by applying nanomedicine. In order to escape from biological barriers, such nanomedicine should be mimicked and designed to be stealthy while navigating in the bloodstream. To achieve this, scientists take advantage of erythrocytes (red blood cells; RBCs) as drug carriers and develop RBC membrane (RBCm) coating nanotechnology. Thanks to the significant advances in nanoengineering, various facile surface functionalization methods can be applied to arm RBCm with not only targeting moieties, but also imaging agents, therapeutic agents, and nanoparticles, which are useful for theranostic nanomedicine. This review focuses on refunctionalization of erythrocyte-based nanomedicine for enhancing cancer-targeted drug delivery.

## Introduction

As a major cause of global deaths, cancer is second only to cardiovascular diseases, which is responsible for nearly 10 million deaths in 2018. Cancer develops in a complex mechanism that stimulates the transformation of a pre-cancerous lesion to a malignant tumor. Cancer cells proliferate rapidly and can spread to other organs of the body (metastases). Thus, early detection and treatment are needed to increase patients' survival and reduce medicinal costs 1, 2. Despite significant improvements in major pillars of cancer treatments, we still find state-specific side effects of chemotherapy or radiotherapy 3.

In response to the above issues, scientists have put tremendous efforts to develop nanomedicine to combat cancer at the molecular level. Nanomedicine exhibits lesser side effects, better delivery efficiency, longer circulation time, and more efficient retention when compared with free drugs 4. This relatively booming research area focuses on nanoparticles to diagnose diseases or, as carriers, to deliver drugs or therapeutic agents 5. Moreover, they can carry therapeutic and diagnostic agents at a single particle, be functionalized with targeting ligands, encapsulate multiple drug molecules, and bypass traditional drug resistance that enhances the accumulation of drugs in cancer cells 4, 6-9.

Despite significant advances in cancer nanomedicine, it still faces biological barriers, including nanoparticle distribution, toxicity, and clearance by the renal system and the mononuclear phagocytic system (MPS). The latter system mediates phagocytic cells to eliminate nanoparticles mainly in the liver and spleen, while the former system ejects nanoparticles with a hydrodynamic diameter of smaller than 5.5 nm in the kidney 10-13. Only those that escape the above-mentioned barriers can interact with tumor tissues. Furthermore, a recent study revealed that only a small amount of the administered nanoparticles (0.7%) is delivered to a solid tumor 14. To tackle this matter, various cell and cell-membrane coating technologies have been developed in building stealth and targeted-nanomedicine. Among them, RBCs possess remarkable properties for drug delivery, such as long blood circulation up to 120 days, ideal for encapsulation of various bioactive compounds (e.g., enzymes, drugs, proteins, and macromolecules), and the most abundant cells in the human body (40-50% of blood volume) 15.

**Figure 1:**
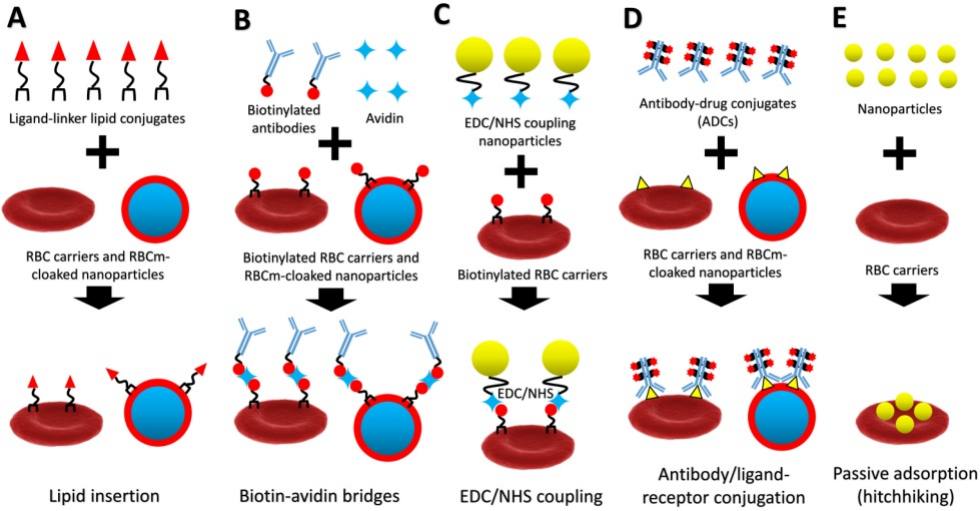
A schematic diagram of lipid insertion (**A**), biotin-avidin bridges (**B**), EDC/NHS coupling (**C**), antibody/ligand-receptor conjugation (**D**), and passive adsorption (hitchhiking) (**E**) methods for refunctionalization of erythrocyte-based nanomedicine.

Recently, many scientists have successfully applied RBCs as carriers for drugs or nanoparticles and RBCm coating for cancer treatment. In fact, many sources of cells can be used for cell membrane coating, where RBCm coating is the popular one because it is easy to isolate and possesses longer life span than the others 16. However, due to lack of cancer-targeting properties of RBCm compared to cancer-membrane coating 17, it requires surface functionalization with targeting moieties to enhance its delivery efficiency to cancer. Therefore, this review focuses on refunctionalization of erythrocyte-based nanomedicine for cancer-targeted drug delivery. We review advanced techniques to engineer RBCm with covalent and non-covalent conjugation methods, including lipid insertion, biotin-avidin bridge, EDC/NHS coupling, antibody/ligand-receptor conjugation, and passive adsorption (hitchhiking) for theranostic purposes (**Figure 1**). These surface functionalization techniques have improved the delivery efficiency of erythrocyte-based nanomedicine in cancer chemotherapy, cancer immunotherapy, cancer photodynamic therapy (PDT)/photothermal therapy (PTT), and their combined therapy. These advances in surface functionalization of erythrocyte-based nanomedicine show great potential for cancer treatment and its clinical translation.

## Erythrocyte-based Nanomedicine in Cancer Drug Delivery

### Engineered RBCs

Engineered RBCs, known as RBC carriers, have been studied since the 1960s as a cell-based drug delivery system, which has transformed from passive carriers into active carriers due to the rapid developments in surface engineering/functionalization of RBCs to target certain diseases. These natural cells possess unique properties that make them capable of carrying therapeutic agents, improving pharmacokinetics, altering pharmacodynamics, and modulating immune responses. Recently, there are two main strategies in utilizing RBCs as drug delivery systems, namely internal loading and surface loading. The latter can be performed both *ex vivo* and *in vivo* 16. To encapsulate drugs in RBCs, several *ex vivo* methods can be used, such as hypotonic pre-swelling, hypotonic dialysis, hypotonic dilution, osmotic pulse/isotonic hemolysis, electroporation, cell-penetrating peptide (CPP), and available red-cell loader technology 17, 18. In addition, smart drug delivery systems are now on the rise of applications in the biomedical fields, especially for cancer and cardiovascular disease treatments 19-31. For instance, one of these systems has been adopted by scientists to built intelligent RBC carriers by transporting nanoparticles on their surface and detaching by a specific stimulus (e.g. shear stress) 16. Further functionalization of natural RBCs can enhance the targeting ability of the engineered RBCs and provide a feasible way to carry nanoparticles or imaging agents for the synergistic combination of multiple therapies and theranostic purposes.

### RBCm-Cloaked Nanoparticles

In addition to the rapid developments of engineered RBCs as drug carriers, some top-down technologies are now being used to leverage RBCm as building substances of nanocarriers 32. Upon removal of the intracellular parts via hypotonic lysis, the obtained membrane vesicles can be used to coat nanoparticles either through extrusion, sonication, or microfluidic electroporation 33, 34. These RBCm-cloaked nanoparticles possess the biocompatibility of the mother cells and its preserved complex proteins allow them to circulate for a long time in the bloodstream 33, 37. The surface glycoproteins, especially the signaling CD47 molecules, inhibit macrophages to phagocyte RBCm-cloaked nanoparticles by transducing an inhibitory “do not eat me” signal 38. Furthermore, this unique biomimetic approach has been widely used in camouflaging synthetic carriers such as polymeric nanoparticles to behave like native RBCs while circulating, possessing longer circulation, due to its unique membrane properties that can regulate the adsorption of biological components, reducing the protein corona formation 35. In cancer drug delivery, a number of publications reported the use of poly(lactic-co-glycolic acid) (PLGA) nanoparticles, elastomeric copolymers that possess excellent properties, including biodegradability and biocompatibility, FDA approvals, and simple-synthesized methods 11, which are camouflaged with RBCm and functionalized with targeting ligands to gain stealthy effects and enhance the specific targeting of the RBCm-cloaked PLGA NPs 39. However, along with the development of active targeted RBCm-cloaked nanoparticles, there are still many studies that use passive targeting via the EPR effect to deliver these biomimetic nanoparticles to solid tumor 40-42.

## Surface Engineering/Functionalization of RBCm

### Lipid Insertion

Chemical synthesis methods (e.g., carboxyl-, amine-, and thiol-based chemistry) have been widely used to functionalize nanoparticles with targeting ligands 43, 44. However, in the case of natural-derived carriers such as RBCs, a non-disruptive functionalization approach is much more desirable than direct chemical conjugations due to the existence of biological molecules on RBCm. Recently, scientists have developed a lipid-insertion approach to functionalize RBC carriers and RBCm-cloaked nanoparticles that prevent RBCm from damages due to the exposure to chemical reactions. This attachment depends on either the alkyl chain or the lipid section of molecules to be inserted into lipid bilayers of RBCm via direct mixing (stirring) for a few minutes or hours at a room or 37 °C temperature. The unbound lipid conjugates can be removed via centrifugation 45, 46. This physical insertion of ligand-linker-lipid conjugates can facilitate RBCm to be functionalized with targeting, imaging, and therapeutic molecules without damaging the existing surface proteins 47. In most cases, detachable poly(ethylene glycol) (PEG)-lipid conjugates are used as ligand-linker-lipid conjugates for cell membrane-surface modification 48. PEG-lipid conjugates incorporating functionalized PEG termini can be easily found in the market (e.g., 1,2-distearoyl-sn-glycero-3-phosphoethanolamine (DSPE)-PEG-maleimide, DSPE-PEG-folate, DSPE-PEG-biotin, DSPE-PEG-amine, and DSPE-PEG-carboxylic acid). DSPE-PEG-folate, for instance, can directly be used as active targeting agents in cancer drug delivery as folate receptors are highly overexpressed on the surface of many tumor types 49. A study revealed that unmodified and folate-modified RBCm-cloaked nanoparticles exhibited similar physicochemical properties, suggesting that this surface modification had minor side effects on the properties of nanoparticles 50. Furthermore, this lipid insertion method allows a relatively large targeting ligand (e.g., MW ~9000 Da) to be incorporated into a smaller lipid anchor (e.g., MW ~748 Da) that facilitates an efficient conjugation with RBCm 47.

### Biotin-Avidin Bridges

Biotin-avidin bridges, which are the most frequent use of indirect surface engineering of RBCm, require direct modification (anchored molecules) on the RBCm via a lipid insertion method or a chemical modification. However, using lipid anchors, such as DSPE-PEG-biotin, to construct biotinylated RBCs or biotinylated RBCm-derived vesicles are less destructive than using chemical modifications, such as NHS-biotin or Sulfo-NHS-biotin. The tetrameric avidin contains four identical subunits, each of which can specifically and strongly bind to biotin. This complex is the strongest known non-covalent interaction (Kd = 10-15 mol/L) between proteins and ligands with a rapid formation and stable bonding (unaffected by extreme pH, temperature, organic solvents, and other denaturing agents) 51. The reaction time of biotin-avidin can be done around 1 h at room temperature, and to fully block the unreacted sites of avidin/streptavidin, incubation of free biotin molecules can be done for another 10 min and washed with PBS to remove the unreacted biotin 52. These biotin-avidin bridges are commonly used in decorating nanoparticles with targeting ligands by facilitating biotinylated nanoparticles and targeting ligands through avidin-linkages 53. For instance, a tumor-targeting peptide c(RGDyK) could be attached to the RBC-coated drug nanocrystals via biotin-avidin bridges followed by a lipid insertion method for targeting glioma and delivering the chemotherapeutic drugs 54. Interestingly, this one of the strongest known non-covalent interaction in nature allows bioengineered motile bacteria, Escherichia coli MG1655, to be conjugated with RBCs to construct bacteria-driven microswimmers for cancer drug delivery and PTT 55, 56.

### EDC/NHS Coupling

As a zero-length crosslinking agent, the 1-ethyl-3-(3-dimethylaminopropyl)-carbodiimide (EDC) mediates the coupling between carboxyl groups and primary amines by forming an amine-reactive *O*-acylisourea intermediate (after a reaction with a carboxyl group of biomolecules) that quickly reacts with an amino group to form an amide bond with release of an isourea by-product 57. Excessive reagents and by-products can be easily cleared by dialysis or gel-filtration methods 58. After EDC is water-soluble, the crosslinking can be performed under normal condition without adding organic solvents, but it requires fast reaction due to the rapid hydrolyzation of the formed reactive ester in aqueous solutions 58, 60. To stabilize this active ester, *N*-hydroxysuccinimide (NHS) or *N*-hydroxysulfoxuccinimide (Sulfo-NHS) can be introduced 59. Importantly, when using EDC we should control the key parameters (pH, the amount of EDC, and the ratio EDC/NHS) to optimize the hydrolysis reaction and prevent nanoparticles from aggregation 60-62. In cell-membrane nanotechnology, this EDC/NHS coupling reaction can be facilitated to prepare indirect conjugation of inorganic nanoparticles on the RBCm because direct conjugation, such as conjugation of peptides/proteins to RBCm using chemical reaction (eg. EDC) may damage RBCm, especially reduce the plasticity 63. Wang et al., for instance, designed upconversion nanoparticles (UCNPs)-painted RBCs for fluorescence imaging-guided tumor surgery and PDT. The hexanoic acid ester-modified rose bengal, RGD peptides, and avidin were covalently coupled onto amine-modified UCNPs via EDC/NHS reaction 64. Similarly, Zhu et al. functionalized tumor-targeting molecule folic acid and magnetic nanoparticles (MNPs) on the surface of RBCs for filtration of circulating tumor cells (CTCs). The carboxyl groups on the surface of MNPs were activated into esters via EDC/NHS reaction, reacted with the amino of streptavidin to form a stable acid amide bond, and conjugated with biotinylated anti-human ter119 antibody using biotin-streptavidin bridges, respectively 65.

### Antibody/Ligand-Receptor Conjugation

As mentioned before, chemical conjugation methods require cell isolation, functionalization, and reinjection that restrict their practical use in clinical translation. By using the antibody/ligand-receptor conjugation method, we can couple therapeutic agents to circulating RBCs *in vivo*, which provide a relatively safe method to evade complications associated with *ex vivo* modification and transfusion 66. Therapeutic agents can be conjugated with antibodies, antibody fragments, peptides, or other ligands that bind to RBCm, which can be performed both *ex vivo* and *in vivo*. The latter approach is performed by intravascular injection of antibody-drug conjugates (ADCs) leading to rapid binding to circulating RBCs in the bloodstream 67. There are several RBCm proteins that are frequently used as targeted receptors for ADCs, namely band 3, glycophorin A (GPA), and complement receptor 1 (CR-1), which account ~1,000,000, ~1,000,000, and ~1,000 copy per single RBC, respectively 66. In addition, a new developed single-chain variable fragment (scFv) of monoclonal antibodies to target the RBC receptors, such as GPA, is on the rise of applications as it does not cause the harmful immune responses compared with the murine monoclonal antibodies. This technique is not only suitable to be used for conjugation of therapeutic agents, but also can be used to conjugate nanoparticles on RBCm as demonstrated by Sahoo et al. who successfully attached copolymer-based nanoparticles encapsulating fluorescent-BSA on the surface of RBCm using different GPA-specific targeting ligands, namely ERY1, scFv TER-119, protamine-based CPP without damaging RBCm. *In vitro* study showed that the protamine-based CPP was superior to the other ligands in binding nanoparticles on RBCm 68.

### Passive Adsorption (Hitchhiking)

Nanoparticles can be functionalized on the surface of RBC carriers via passive adsorption (non-covalent interaction), which uses either hydrogen bonds (dipole-dipole interactions), electrostatic interactions (charge-charge interactions), van deer walls forces, or hydrophobic interactions. However, the binding strength of this technique is much weaker when compared to covalent conjugations. Recently, this passive adsorption has been used to deliver theranostic-based nanoparticles via transportation on the surfaces of RBCs, commonly referred to as RBC-hitchhiking, which can prolong their circulation 69. This strategy was first developed by Chambers and Mitragotri in 2004 motivated by mammalian pathogens (hemobartonella and eperythrozoonosis; diameter: 0.2-2 µm) that bind to the RBCm and remain in circulation for several weeks 70, 71. They decorated RBC surfaces with either 100 nm-, 220 nm-, 450 nm-, 830 nm-, or 1100 nm- polystyrene nanoparticles. The 220 nm- and 450 nm- particles exhibited the longest circulation times (> 7 h) compared to larger particles that were rapidly removed from circulation (< 2 h). In the latter study, polystyrene nanoparticles eventually detached from RBCs due to high shear forces (around 50% at shear stress 10 Pa) and cell-cell interactions (around 70% particles at 50% hematocrit) and are subsequently cleared in the liver and spleen 72. In cancer drug delivery, this strategy was adopted by Zelepukin et al. who painted the surface of doxorubicin (DOX)-loaded RBCs with positively charged chitosan-coated nanoparticles (CTSs). These CTSs were tightly bound to RBCs and exhibited almost no desorption from the cell surface during the washing steps due to the electrostatic interaction. Treatment with these RBC-bound nanoparticles could significantly suppress pulmonary metastatic melanoma that resulted in a 3-fold decrease in the total number of metastatic nodes and an obvious reduction in the average size of nodes *in vivo* 69.

## Applications of Erythrocyte-Based Nanomedicine

### Cancer Targeting

**Figure 2:**
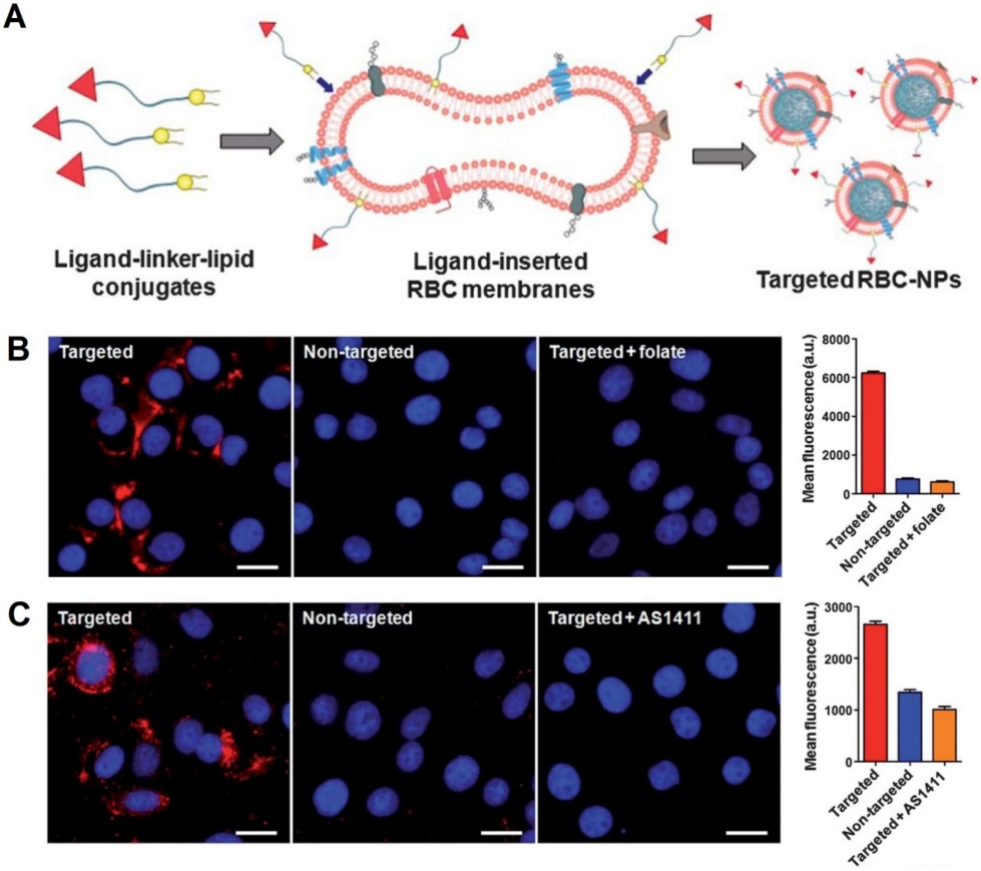
Schematic of the preparation of RBC-NPs with targeting ability. Ligand-linker-lipid conjugates are synthesized and then inserted into RBCm ghosts (**A**). The resulting ligand-functionalized RBCm are used to coat polymeric cores to form targeted RBC-NPs. Fluorescence microscopy images and quantification of the mean fluorescence intensity of flow cytometry histograms of KB cells incubated with folate-functionalized RBCNPs, non-targeted RBC-NPs, and folate-functionalized RBC-NPs together with free folate and AS1411-functionalized RBCNPs, non-targeted RBC-NPs, and AS1411-functionalized RBC-NPs together with free AS1411 aptamer (**B, C**). Adapted under the terms of the CC BY-NC 3.0 license 47. Copyright 2013, the Authors. Published by The Royal Society of Chemistry.

Recently, significant strategies have been proposed to improve cancer-targeting abilities of engineered RBCs and RBCm-cloaked nanoparticles. Fang et al. demonstrated the successful decoration of RBCm-cloaked PLGA nanoparticles (RBC-NPs) using two differently sized ligands, a small molecule FA (MW ~441 Da) and a nucleolin-targeting aptamer AS1411 (MW ~9000 Da) via a lipid insertion technique (**Figure 2A**). *In vitro* results showed that fluorescence signals from the RBC-NPs were significantly seen in folate and aptamer-functionalized RBC-NPs-treated cancer cells and not significantly noticeable in folate and aptamer-negative cancer cells (both flow cytometry and fluorescence imaging observation) (Figure 2B, C) 47. This lipid insertion method has been further utilized by Zhang et al. and Chen et al. who decorated RBCm-cloaked PLGA nanoparticles with bispecific recombinant protein anti-EGFR-iRGD, containing both tumor-penetrating peptides (RGDs) and EGFR single-domain antibodies (sdAbs) to deliver antitumor drug gambogic acid (GA) and paclitaxel (PTX), respectively 73, 74. Interestingly, Zhou et al. developed RBCm-cloaked PLGA NPs that were functionalized with NHS-PEG-maleimide and subsequently conjugated with thiolated human recombinant hyaluronidase, PH20 (rHuPH20) through thiol-maleimide reaction (two-step conjugation). The long linker of NHS-PEG-maleimide (MW ~3400 Da) could maintain enzyme activity more efficiently than the short linker (MW ~425 Da) without significant changes of RBCm stability. The modified rHuPH20 NPs could assist NP diffusion more efficiently than free rHuPH20 to the site of action *in vitro* 75.

Another application of engineered RBCs is to target CTCs. CTCs escape from main tumors and migrate to other organs in the circulatory system, so it is crucial to selectively scan and isolate CTCs to prevent its metastases to different organs. However, recently developed capturing methods of CTCs (e.g., magnetically activated cell sorting (MACS)) still face a serious issue due to high impurities of leukocytes in the sorting process, so we need to develop new strategies that can efficiently quarantine CTCs. Mukthavaram et al. proposed an efficient capturing technique of CTCs using RBCs functionalized with rituximab antibodies via a lipid insertion method (the antibodies were either directly conjugated to the lipid or immobilized via anti-Fc antibody). This functionalization did not disrupt the RBCs as proven by no significant hemoglobin release and aggregation of RBCs. *In vitro* study showed that these engineered RBCs efficiently depleted CD19^+^/CD20^+^/CD45^+^ human lymphoma cells (over 90%) in mantle cell lymphoma (MCL) JeKo-1 models. This was due to the powerful binding effect of rituximab antibodies with cancer cells, forming rosettes. However, the depletion mechanism of CTCs still needed to be further studied. The damaged (distorted) RBCs may be eliminated by the liver kupffer cells through scavenger receptors or enhanced recognition of RBC rosettes through immunoglobulin and complement receptors 76, 77. In another study, Zhu et al. used the tumor-targeting folic acid and magnetic nanoparticles (MNPs) that were conjugated on the surface of RBCs by hydrophobic interaction and chemical conjugation, respectively. The resulting engineered RBCs could rapidly adhere to CTCs and make CTC-RBC complexes, which were then isolated using a magnetic field. The average capturing efficiencies of HCT116 and MCF-7 cancer cell lines were approximately 93.92 ± 3.92% and 93.49 ± 3.03%, respectively, indicating that engineered RBCs could capture CTCs with high efficiency and purity (>75%). The above method was superior to MACS® beads, which only showed 80% for capturing efficiency and 20% for its purity under the same condition 67.

### Cancer Imaging

**Figure 3:**
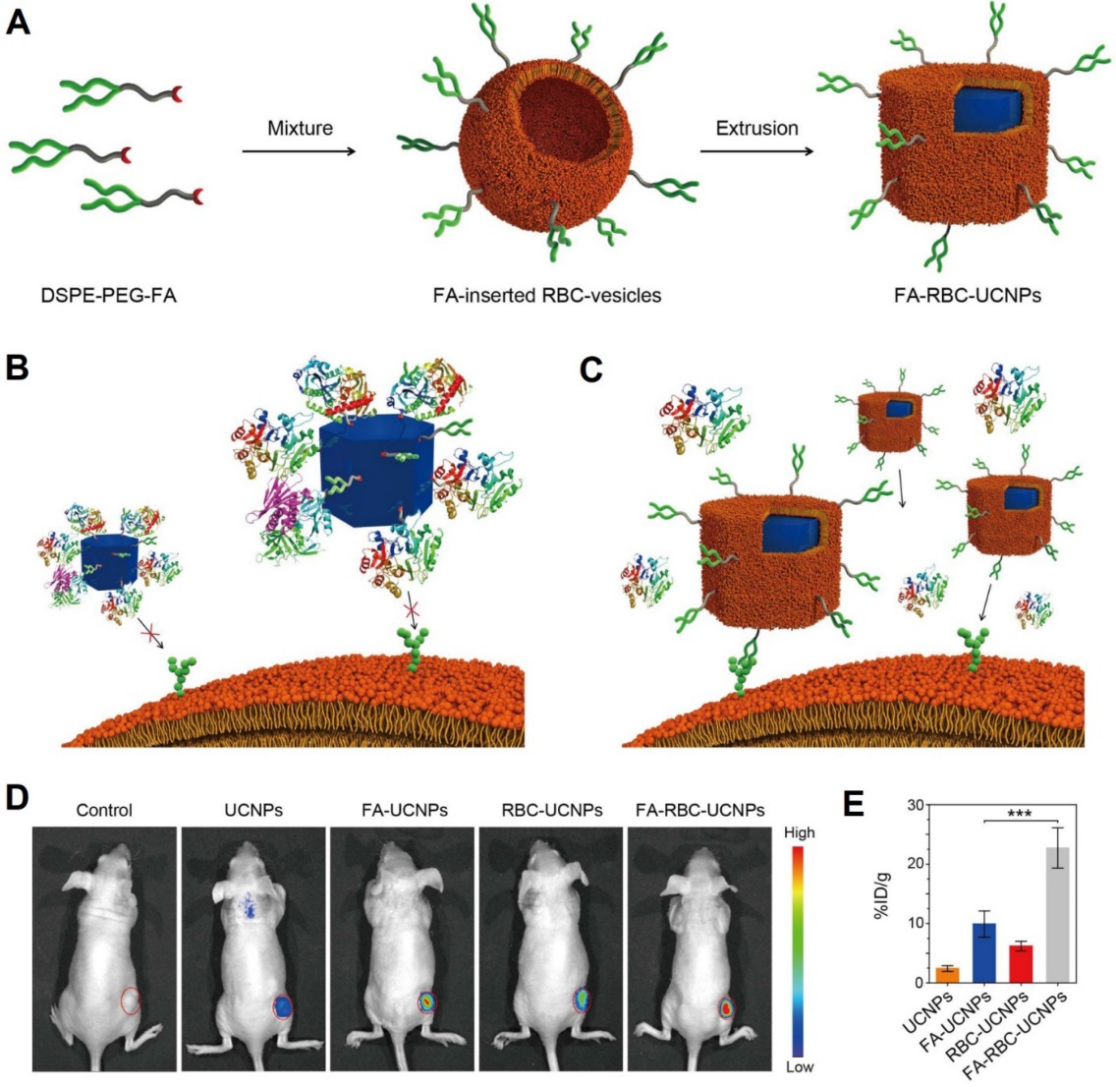
Schematic of preparation of FA-RBC-UCNPs (**A)**. Illustrations of uncoated UCNPs that are surrounded by protein coronas losing their ability to target cancer cells (**B**), and cell membrane-coated nanoparticles retaining its cancer targeting capabilities in a biological environment (**C**). *In vivo* UCL photos of the tumor-bearing nude mice at 48 h after being i.v. injected with PBS or PBS containing various nanoparticles. Red circles: tumor sites (**D)**. Nanoparticle content in the tumor sites at 48 h after the injection. Error bars: standard deviations (*n* = 6) (**E)**. ***: *P* < 0.001. Adapted with permission 51. Copyright 2017, American Chemical Society.

In cancer imaging, both engineered RBCs and RBCm-cloaked nanoparticles have been applied to improve the image quality of molecular imaging modalities (e.g., fluorescence imaging, magnetic resonance imaging (MRI), and photoacoustic imaging (PAI)). Rao et al., for instance, engineered the surface of RBC-UCNPs with folic acid using a lipid insertion method for cancer imaging (**Figure 3A**). The UCNPs can convert light from the near-infrared (NIR) range to the visible range, which is promising for *in vivo* fluorescence imaging. *In vitro* results showed that the folic acid-modified RBC-UCNPs (FA-RBC-UCNPs) could prevent protein corona formation (aggregation) after being exposed to 100% human plasma compared to bare UCNPs (Figure 3B, C). This was due to the RBCm coating possesses natural RBCm's properties that can regulate protein adsorption 35. The unmodified and FA-RBC-UCNPs showed similar physicochemical properties, suggesting that the surface modification of DSPE-PEG-folic acid had minor influences on the properties of UCNPs. *In vivo* results showed that FA-RBC-UCNPs exhibited the brightest green upconversion luminescence (UCL) at the tumor site both *in vivo* and *ex vivo* (Figure 3D, E), suggesting efficient targeting ability of FA-RBC-UCNPs to the MCF-7 tumor xenografts 50.

Recently, perfluorocarbon nanodroplets have been investigated as a new developed PAI contrast agent. However, due to the limited space for drug encapsulation and many drugs are not soluble in perfluorocarbon, so there is a growing interest in searching a new method to engineer these perfluorocarbon nanodroplets. One of the possible methods is to attach perfluorocarbon nanodroplets on the surface of RBCs. This method was adopted by Dixon et al. who loaded RBCs with ICG and 50-nm iron oxide nanoparticles (IONs) via hypotonic dilutional hemolysis and further functionalized RBCs with perfluorocarbon nanodroplets to make acoustically active RBCs (aaRBCs). The nanodroplets were attached into RBCs' surface via electrostatic interaction. *In vivo* results showed that over 80% of the injected aaRBCs remained in the bloodstream with very little accumulation in tumor tissues. This indicated that active targeting was needed to effectively deliver drugs to the tumor sites. Interestingly, the PAI allowed aaRBCs for imaging without destroying RBCs, suggesting their potential for theranostic nanomedicine 78.

Ding et al. recently developed an exosome-like nanozyme vesicle for *in vivo* H_2_O_2_-responsive PAI of nasopharyngeal carcinoma. The intrinsic peroxidase-like activity of graphene quantum dot nanozyme (GQDzyme) could effectively convert the 2,2'-azino-bis (3-ethylbenzothiazoline-6-sulfonic acid) (ABTS) into its oxidized form upon exposure to H_2_O_2_, leading to strong NIR absorbance. Biomimetic strategy using RBCm with folate targeting (DSPE-PEG2000-FA) could enhance its targeting to nasopharyngeal carcinoma with strongest fluorescence intensity in the tumor site (8x) than in other organs 79.

### Cancer Theranostics

#### Chemotherapy

In cancer chemotherapy, a number of functionalized RBCm-cloaked nanoparticles have recently been developed. Fu et al., for instance, demonstrated an effective approach to co-encapsulate hydrophilic and hydrophobic anticancer drugs (PTX and DOX) into magnetic *O*-carboxymethyl-chitosan (CMC) nanoparticles (CNPs), which further camouflaged with RBCm and functionalized with Arg-Gly-Asp (RGD) peptides. Briefly, PTX and hydrophobic Fe_3_O_4_ nanocrystals were co-encapsulated into CNPs with O/W/O double emulsion and temperature-programmed solidification methods. The combined DOX could be then efficiently adsorbed into CNPs via the porous structure (pore size: ~10.35 nm) and electrostatic interaction, which were subsequently camouflaged with RBCm by an extrusion method, while a lipid insertion method facilitated the RGD peptides to be inserted on the RBCm-cloaked nanoparticles. The particle size and zeta potential of these nanoparticles were nearly 140 nm and -10 mV, respectively. *In vitro* results showed that this nanotherapy exhibited tumor growth inhibition approximately 6-fold greater than commercial free Taxol^®^ and DOX after 21 days and 5-fold greater than the control group after 14 days 80.

Recently, the use of PLGA nanoparticles with camouflaged RBCs have been explored in cancer chemotherapy. Luk et al., for instance, synthesized RBCm-PLGA nanoparticles to deliver DOX in lymphoma-model mice. These nanoparticles had a particle size and zeta potential of approximately 155 nm and -30 mV, respectively. However, this formulation failed to diminish tumor and only prolong the tumor growth inhibition, which might be due to the lack of active targeting 81. In another study, Zhang et al. tried to modify the surface of RBCm-PLGA NPs with the anti-EGFR-iRGD recombinant protein via a lipid insertion method to construct iE-RBCm-PLGA NPs and assess their active tumor-targeting ability in colorectal cancer models. In addition, potential antitumor drug GA was loaded into the nanosystem for chemotherapy. The hydrodynamic diameter of iE-RBCm-GA/PLGA NPs was about 153 ± 3.83 nm, which was stable for a week. This nanotherapy could reduce the side effects of GA, prolong tumor inhibition, and ensured safety *in vivo* 73. Similar work was done by Chen et al. who load the anti-EGFR-iRGD modified-RBCm/PLGA NPs with PTX (denoted as PRP). *In vitro* characterization showed that this PRP was spheroid, uniform in size (~171.7 ± 4.7 nm), and stable for 8 days. The encapsulation and drug loading efficiency of PRP were 60.00% and 34.07%, respectively. *In vivo* studies showed that PRP accumulated in tumor sites within 2 h of administration, had long circulation (48 h), and reduced the tumor volume by 61% without causing severe adverse effects. 74.

In other work, Fu et al. designed RBCm-coated solid lipid nanoparticles-based dual-modified T7 and NGR peptides (T7/NGR-RBCSLNs) encapsulating vinca alkaloid vincristine drug to treat glioma. T7 was functionalized to help nanoparticles cross the blood-brain barriers (BBB), while NGR was specifically used to target glioma. The conjugation techniques of both ligands are using a lipid insertion method through two steps. DSPEPEG_2000_-maleimide was firstly conjugated to the cysteine residues of T7 and NGR to synthesize DSPE-PEG_2000_-T7 and DSPE-PEG_2000_-NGR, respectively. The modified ligands were then conjugated to the RBCm by stirring for several hours. *In vivo* study revealed that these biomimetic nanoparticles could significantly prolong the survival rate of mice compared to free drug treatment 82. A similar study used c(RGDyK) peptide-functionalized RBCm-coated drug nanocrystals encapsulating docetaxel (RGD-RBC-NC(DTX)) to treat glioma. This biomimetic nanotherapy showed much higher accumulation in tumor area than in major organs, significantly inhibited tumor progression, and improved the survival rate *in vivo* compared to free the drug treatment 54.

#### Cancer Immunotherapy

**Figure 4:**
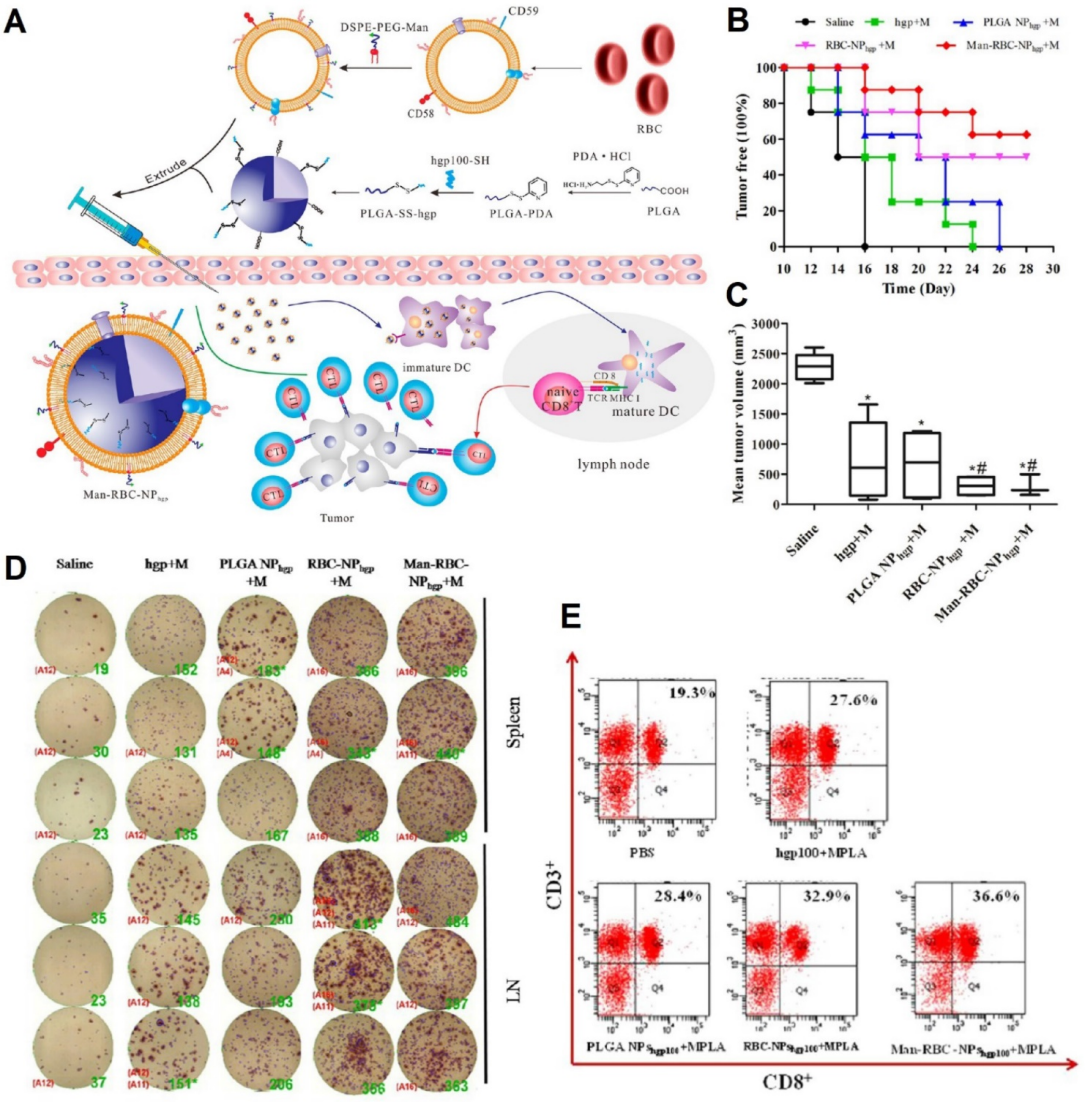
Schematic illustration of the preparation of Man-RBCm-coated PLGA-SS-hgp100 nanoparticles (Man-RBCNP_hgp_) and induction of antitumor immunity (**A**). Efficacy of Man-RBC-NP_hgp_ for tumor occurrence *in vivo*, including the percentage of tumor free mice after tumor challenge (**B**) and the tumor volume at day 28 after tumor cell challenge (**C**). Significant difference vs saline group (*, *p* < 0.05). Significant difference vs hgp+M group (#,* p* < 0.05). IFN-γ production from immunized mice using the ELISPOT assay (**D**). Cytotoxic T lymphocyte (CTL) response after vaccination in tumor-bearing mice (**E**). Adapted with permission 84. Copyright 2015, American Chemical Society.

Manipulating human protection systems (immunity) to recognize and demolish cancer cells is the focus of cancer immunotherapy. Recently, scientists have developed nanovaccines to specifically deliver antigens to antigen-presenting cells (APCs), especially dendritic cells (DCs), which can induce a protective response of antigen-specific T cells. To activate T cells, DCs must migrate to the proximal draining lymph nodes (LNs) after recognizing tumor antigens that promote the secretion of pro-inflammatory cytokines 83. These RBC-based nanovaccines have been integrated to function as immune cells' activation to combat cancer cells. Gue et al., for instance, developed RBCm-PLGA nanoparticles encapsulating antigenic peptide (hgp100_25-33_) and monophosphoryl lipid A (MPLA), which were functionalized with actively targeted mannoses via a lipid insertion method (ManRBC-NP_hgp_+M) (**Figure 4A**). The engineered nanovaccines could specifically target APCs in the lymphatic organ and the redox-sensitive linkers would be cleaved in the intracellular milieu, resulting in the release of antigens. These nanovaccines not only could prolong tumor-occurring time, inhibit tumor growth by 76.6%, and suppress tumor metastasis in prophylactic, therapeutic, and metastatic melanoma models, but also could enhance IFN-γ secretion and CD8^+^ T cell response (Figure 4B-D) 84.

Recently, Sun et al. developed pMHC-1, aCD28, and IL2-modified RBCs (R-aAPC-IL2) by engineering antigen peptide-loaded major histocompatibility complex-I and CD28 activation antibodies on RBCs' surface via biotin-avidin bridges, which are further tethered with interleukin-2 (IL2) as a proliferation and differentiation signal via a lipid insertion method. The microvaccines could not only provide a flexible cell surface with appropriate biophysical parameters, but also mimic the cytokine paracrine delivery. These microvaccines mimicked mature DCs, which their CD28, pMHC-1, and IL2 could facilitate the proliferation of antigen-specific CD8^+^ T cells and increase the secretion of inflammatory cytokines. *In vitro* studies showed that no obvious cell death was observed for the two types of cancer cells after getting the treatments with naive splenocytes without activation by R-aAPC-IL2. The activated splenocytes could effectively kill antigen-positive cancer cells via an antigen-specific manner 52.

#### Photodynamic/Thermal Therapy

Both PDT and PTT have shown significant progress in preclinical and clinical studies of cancer treatment. PDT is a treatment based on the ability of photosensitizers to absorb light energy and to transfer it to oxygen molecules thereby forming singlet oxygen (^1^O2) and other noxious reactive oxygen species (ROS) 85. Meanwhile, PTT utilizes photothermal agents to absorb light and induce elevated temperature, leading to local hyperthermia (42-46 °C) or even photothermal ablation (>43 °C for hours or >54 °C for seconds) causing cancer cell necrosis 86.

**Figure 5:**
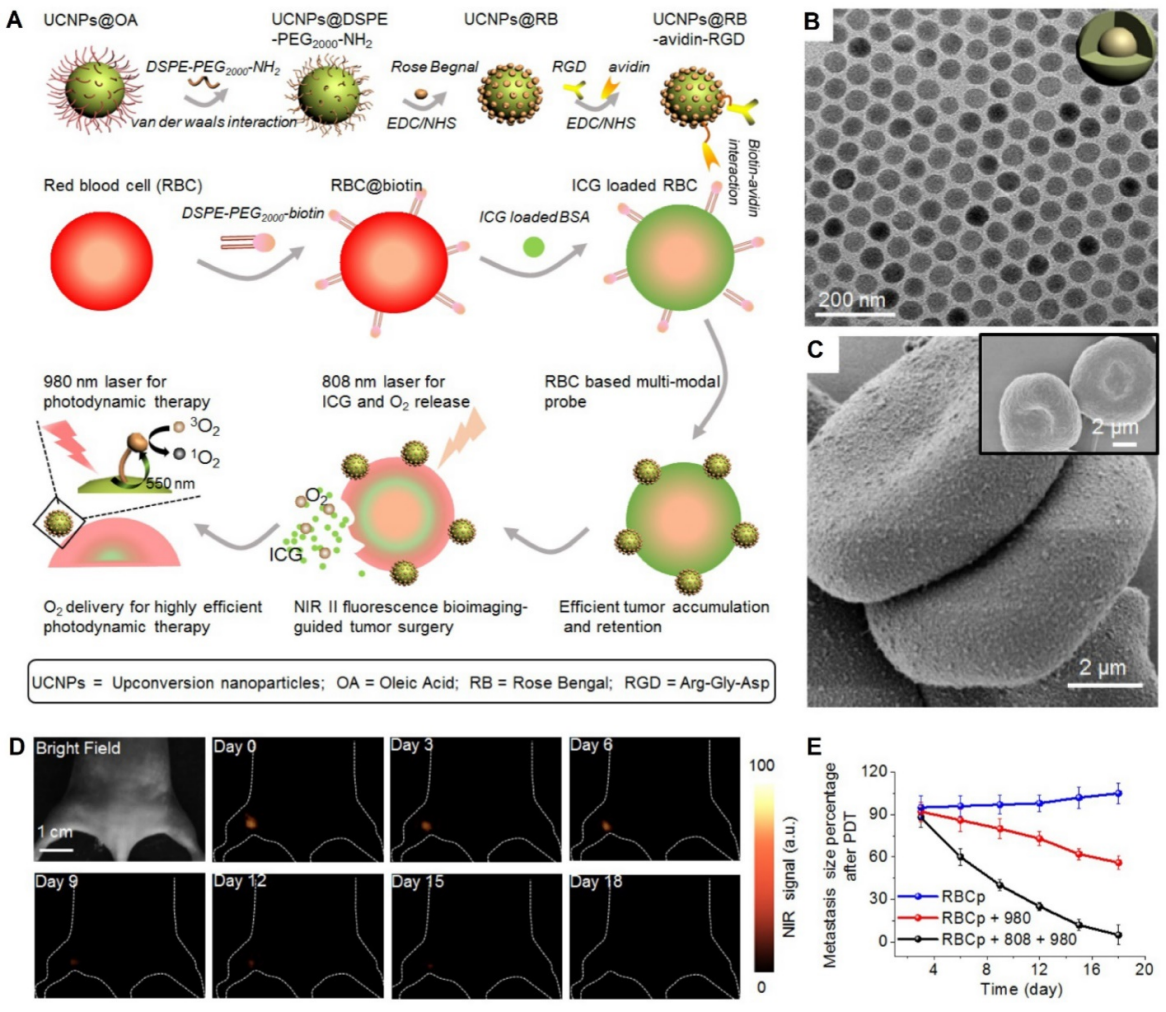
A schematic illustration of RBCp fabrication. With an 808-nm laser irradiation, RBCp can continuously release ICG and O_2_, which can be applied for NIR II fluorescence bioimaging-guided tumor surgery as well as to enhance NIR II fluorescent bioimaging-guided PDT (**A**). TEM image of NaGdF_4_:Yb,Er@NaGdF_4_ with the core-shell nanostructure (**B**). SEM images of two RBC-based multimodal probes attached with UCNPs and RBCs without UCNPs modification (inserted) (**C**). NIR II fluorescence bioimaging-guided PDT of popliteal lymph node metastasis-bearing mice by RBCp injection and alternate irradiation with 808-nm and 980-nm laser (**D**) amd popliteal lymph node metastasis growth profiles after a quantitative NIR II fluorescence analysis of all images (**E**). Adapted under the terms of the CC BY-NC 4.0 license 64. Copyright 2019, the Authors. Published by Ivyspring International Publisher.

Wang et al. constructed engineered RBC-based PDT probes (RBCp) by using lanthanide-doped NaGdF_4_:Yb,Er@NaGdF_4_ nanocrystals (UCNPs) as photosensitizers. The UCNPs were attached on RBC carriers using EDC/NHS coupling and biotin-avidin bridge strategies (**Figure 5A**). The nanoprobes had 40-nm uniform-sized particles that spread evenly on the surface of ICG-loaded RBCs (Figure 5B, C) and 550-nm upconverting luminescence under 980-nm laser irradiation to trigger photosensitization for singlet oxygen generation. *In vivo* results revealed that treatment with RBCp exhibited the highest antitumor efficacy by remarkably inhibiting tumor volumes by 90% over 18 days compared to other treatments (Figure 5D, E) 64.

Recently, Jiang et al. fused RBCm together with MCF-7 cell membranes and fabricated erythrocyte-cancer hybrid membrane-camouflaged melanin nanoparticles (Melanin@RBC-M) for cancer PTT. RBCm coating increased the hydrodynamic size of melanin nanoparticles 216 nm to 240 nm and changed the zeta potential of nanoparticles from -25.2 ± 0.5 mV to -31.1 ± 0.8 mV 87. Several studies reported that surface antigens were responsible for the homologous adhesion nature of cancer cells, and the cancer-cell-membrane coating enhanced cancer-targeting ability 88. Melanin@RBC-M significantly enhanced the red fluorescence signals in MCF-7 cells compared with 4T1, MCF-10A and RAW264.7 cells *in vitro*, suggesting the specific targeting of Melanin@RBC-M towards MCF-7 cells. The treatment of Melanin@RBC-M in mice exhibited a significant rise of tumor temperature from 29.6 °C to 54.0 °C within 10 min with 100% tumor elimination, which demonstrated its effective tumor ablation ability *in vivo* 87.

Ding et al. developed UCPNs-based RM-coated biomimetic PDT agents with folate (DSPE-PEG_2000_-FA) and triphenylphosphonium cation (DSPE-PEG_2000_-TPP) decorated on the surface for *in vivo* PDT treatment of tumors. The hydrophobic UCNPs were entrapped in the hydrophobic core of triblock copolymer polypropylene oxide (PPO)-polyethylene oxide (PEO)-PPO forming nanocarriers. The targeting moieties were inserted into the phospholipid layer of erythrocytes' membrane during the process of extrusion. *In vivo* study revealed that PDT using these NPs could suppress the progression of tumor with the longest survival rate compared to the other treatments 89.

#### Combined Therapy

In recent years, combination therapies have shown great promise in fighting cancer. Commonly, PDT/PTT is combined with chemotherapy. Alapan et al., for instance, constructed bacteria-driven microswimmers using RBCs as autologous drug carriers for active and guided drug delivery to treat tumor via combined chemo and PTT. These biohybrid microswimmers were fabricated by attachment of bioengineered motile bacteria, Escherichia coli MG1655, on SPIONs-DOX-loaded RBCs via TER-119 antibody-anchored biotin-avidin-biotin binding complex. The bacteria autonomously guided RBCs with the help of external magnetic guidance from SPIONs using a uniform magnetic field (20 mT). Interestingly, RBC microswimmers displayed high stability and deformability when squeezing through the constricted channels due to its strong noncovalent conjugation between the bacteria and RBCs. Furthermore, on-demand NIR-activated hyperthermia was performed to eliminate bacteria after cargo delivery. The encapsulation efficiency of DOX in RBCs was around 78%. *In vitro* drug release showed that around 98% of DOX molecules were released at pH 3.1 within 24 h, suggesting its potential for cancer drug release. The mean speed of RBC microswimmers was 10.2 ± 3.5 µm/s, whereas free bacteria displayed a mean speed of 19.5 ± 9.2 µm/s. For PTT purposes, indocyanine green (ICG)/BSA complex was loaded in RBC microswimmers, and NIR irradiation resulted in the rupture of RBCm due to the effects of hyperthermia (>60 °C) leading to the death of attached bacteria 55. These RBC microswimmers exhibited higher average velocities compared with previous designs using synthetic cargo carriers and chemical attachment methods 90. ICG is a potent photothermal and FDA-approved NIRF imaging agent for cancer theranostics. Recently, Li et al. developed two-dimensional graphene oxide core nanoparticles encapsulating ICG and DOX coated with RBCm and functionalized with targeting ligands and imaging agents (F-RGID). To functionalize the RBCm, folic acid and fluorescein isothiocyanate (FITC) were conjugated to DSPE-PEG_2000_-NH_2_ via EDC/NHS coupling, and then the resulting targeting ligands and imaging agents were fused with RBCm via the lipid insertion method. F-RGID had hydrodynamic size and zeta potential of approximately 170 nm and -28 mV, respectively. *In vivo* PTT study showed that F-RGID could increase the temperature in tumor area from around 32 °C to 48 °C after laser irradiation at 808 nm with intensity of 2 W/cm^2^ for 300 s, which effectively reduced the tumor volume 91.

**Figure 6:**
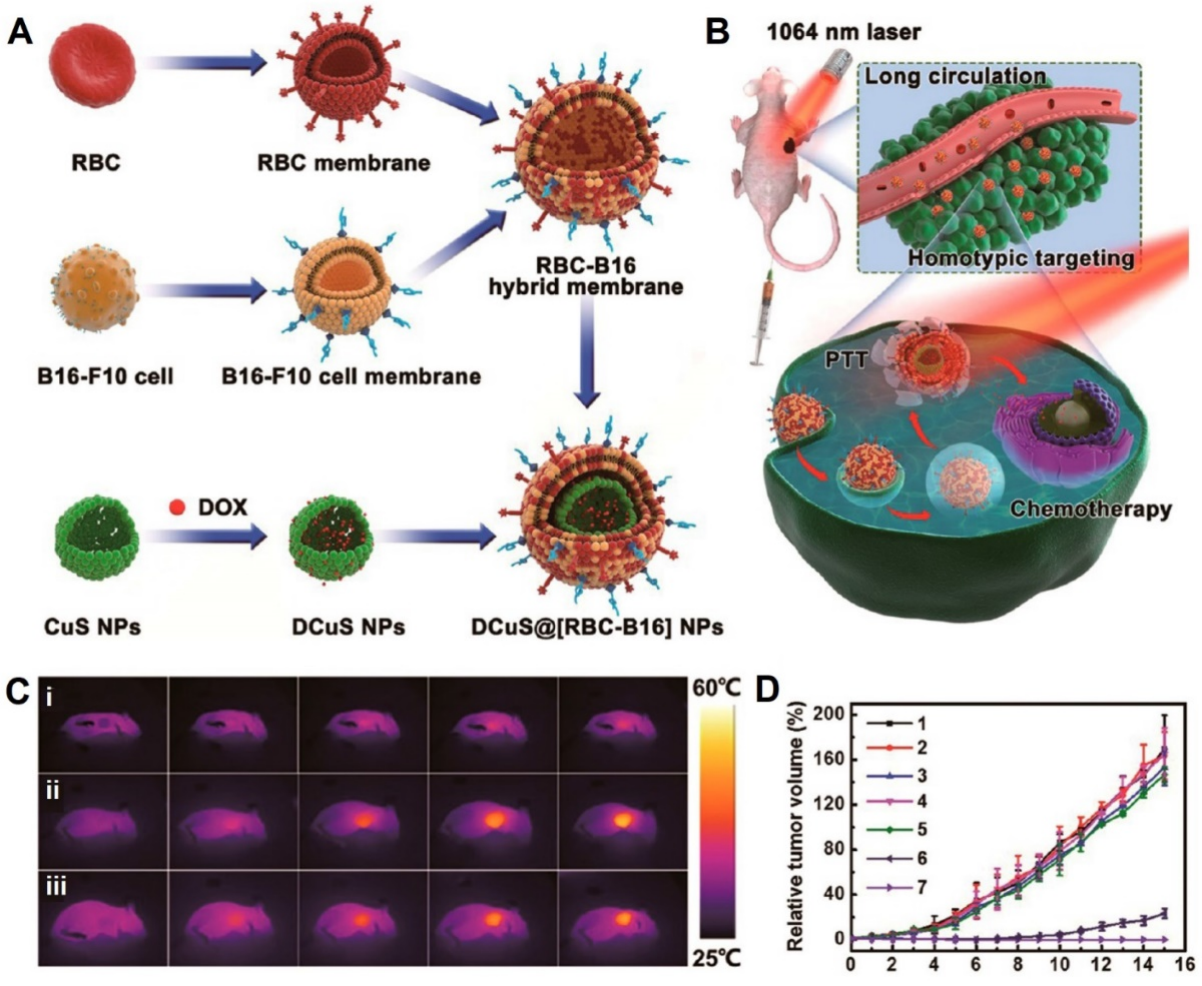
Schematic of membrane fusion and coating. Membrane materials are derived from RBCs and B16-F10 cells and then fused together. The resulting hybrid membrane is used to camouflage DOX-loaded hollow copper sulfide nanoparticles (DCuS NPs) to produce DCuS@[RBC-B16] NPs (**A**).Synergistic photothermal/ chemotherapy of melanoma (**B**).Infrared thermography of tumor-bearing mice that were tail vein injected with (i) normal saline, (ii) CuS@[RBC-B16], and (iii) DCuS@[RBC-B16] (**C**).Relative tumor volume of melanoma-bearing mice receiving different treatments (1: normal saline, 2: CuS@[RBC-B16], 3: DOX, 4: NIR laser (1,064 nm, 1 W/cm^2^), 5: DCuS@[RBC-B16], 6: CuS@[RBC-B16] with NIR laser (1,064 nm, 1 W/cm^2^), 7: DCuS@[RBC-B16] with NIR laser (1,064 nm, 1 W/cm^2^) (**D**). Adapted with permission 70. Copyright 2018, American Chemical Society.

Wang et al. fused RBCm and melanoma cell (B16-F10) membranes (RBC-B16) to coat DOX-loaded hollow copper sulfide nanoparticles (DCuS@[RBC-B16] NPs), which showed potential for synergistic PTT/chemotherapy of melanoma (**Figure 6A**). The loading capacity and loading efficiency of DOX were around 87.7% and 95.5%, respectively. The average size and zeta potential of DCuS@[RBC-B16] NPs were about 220 nm and -23 mV, respectively. The treatment of DCuS@[RBC-B16] NPs in mice exhibited a significant temperature rise up to 51.2 °C under the 1064-nm NIR laser irradiation for 5 min, suggesting hyperthermia effects combined with chemotherapy to synergistically kill the cancer cells with almost 100% of tumor inhibition *in vivo* (Figure 6B-D) 92. Similarly, Sun et al. developed RGD-functionalized RBCm-cloaked NPs loading ICG-BSA complex and DOX (IB&D@RBC-RGD) for the combination of PTT and chemotherapy. IB&D@RBC-RGD showed an increased temperature from ~34 to ~50 °C upon 808-nm laser irradiation of 0.5 W/cm^2^ for 5 min and exhibited ~80% of DOX release for 10 min with the same intensity of laser irradiation 93.

Wang et al. functionalized DOX-loaded RBCs with chlorin e6-coated IONs via biotin-avidin bridges. The surface of the IONs was PEGylated, obtaining a DOX@ RBC-IONs-Ce6-PEG structure with long blood-circulation and strong responses to an external magnetic field. The combination therapy using DOX@RBC-IONs-Ce6-PEG exhibited the most potent cancer cell elimination *in vitro*. The combination therapy with magnetic targeting demonstrated the strongest tumor-growth inhibition effect (67.0%) compared to chemotherapy or PDT alone, which only showed around 44.7% and 35.4% of tumor growth inhibition, respectively 94.

Interestingly, PTT can be combined with immunotherapy. Very recently, Liang et al. synthesized biomimetic black phosphorus quantum dots (BPQDs-RMNV) to kill breast cancer cells by NIR irradiation-mediated PTT to mobilize the immune system for complete elimination of the residual and metastatic cancer cells. *In vitro* studies study showed that breast tumor cell (4T1) lines underwent apoptosis and necrosis after NIR laser irradiation and recruited DCs to capture the tumor antigens *in vivo*. Furthermore, programmed cell death protein 1 antibodies (aPD-1s) were employed to prevent the CD8^+^ T cells from exhaustion. The average diameter and zeta potential of BPQDs-RMNV were approximately 100 nm and -13 mV, respectively. The photothermal effect of the BPQD-RMNVs could eliminate cancer cells by more than 90% with subsequent recruitment of CD11c^+^ DCs. Furthermore, mice treated with BPQD-RMNVs+laser+aPD-1 and BPQD-RMNVs+laser showed an increased number of T cell population 4- to 5-fold higher than the control group. These results demonstrated that BPQD-RMNV-mediated PTT combined with aPD-1 significantly destroyed cancer cells and eliminated the residual and metastatic tumor growth *in vivo* 95.

Interestingly, Song et al. developed RBCm-cloaked nanogels for chemotherapy and immunotherapy of cancer. Nanogels were constructed from amphoteric methacrylamide *N*-carboxyethyl chitosan (CECm) and positive charged methacrylamide *N*-(2-hydroxy)propyl-3-trimethylammonium chitosan chloride (HTCCm) and loaded with PTX. After coating with RBCm, immunotherapeutic agent interleukin-2 (IL-2) was decorated on the surface of NPs via binding to the IL-2 receptor (glycoprotein) on the RBCm to form PTX and IL-2 co-loaded NR (NR_P+I_). This NR_P+I_ could absorb 89.3 ± 5.4% of IL-2 from the given IL-2 on its surface. DLS measurements showed that this NR_P+I_ had a size and zeta potential of around 336.0 ± 6.2 nm and -7.1 ± 0.6 mV, respectively in pH 7.4. *In vitro* study showed that only 8.7 ± 0.3% of adsorbed IL-2 was released from NR_P+I_ within 24 h in pH 7.4. 55.2 ± 2.6% of IL-2 was rapidly released from NR_P+I_ within 8 h in pH 6.5. In pH 7.4, only 31.7 ± 2.8% of entrapped PTX was released in NR_P+I_ over 24 h, which suggested that RBCm may serve as the protective layer and prevent the burst release of PTX from the naked NG core. When tested in simulated acidic tumor microenvironment (pH 6.5), NR_P+I_ presented rapid PTX release behavior about 73.6 ± 4.8% during 24 h 96.

## Conclusions and Future Perspectives

Cancer still tops the list as a leading cause of global deaths. Its treatments with a practically medieval method of surgery, radiotherapy or chemotherapy still abandon adverse effects with high probabilities of recurrence and propagation to other organs 1. The slow progress of cancer treatments results in a poor success rate of clinical trials 97. Nowadays, molecular-targeted therapies become an integral part of new cancer treatments 98, 99. Significant advances in cancer genomics have shifted the old paradigm of treatments, giving birth of “tailored therapy” that applies the targeted therapies based on individual identified therapeutic targets.

To implement this, nanotechnology has been used to develop more efficient and safe nanomedicine with targeted ligands, imaging molecules, and therapeutic agents (e.g., drugs, genes, proteins or enzymes, etc.), which offer theranostic hallmarks and specific cancer targeting to synergistically combat cancer. Furthermore, nanotechnology not only facilitates chemo-drug delivery, but also offers newly developed treatments (e.g., gene delivery, PDT and PTT, cancer immunotherapy, and combined therapy). However, to escape from biological barriers, such agents should be mimicked and designed to be stealthy while navigating in the bloodstream and finally reaching the cancer site via ligand/antibody-receptor axis.

Recently, scientists have developed a number of engineered RBCs and RBCm-cloaked nanoparticles. To improve its specific targeting, the surface of RBCm can be further functionalized using various techniques, either covalent or non-covalent conjugation. In addition, fusion with cancer cell-membrane itself (hybrid cell membrane coating) can also enhance its specific targeting to cancer cells. Due to the presence of biological components on RBC surfaces or RBCm vesicles, it demands a non-disruptive functionalization strategy. Recently, scientists have developed a lipid insertion method by physically inserting ligand-linker-lipid conjugates into the RBCm to produce functionalized RBCm without damaging the existing surface proteins 47. This physical insertion method is quite simple, does not take a long time and high temperature for reaction and disrupt the RBCm. However, the conjugation may not be as strong as chemical conjugation upon applied forces, affecting the attached molecules to detach. Moreover, free proteins in the bloodstream may pull out the lipid anchors from RBCm due to their hydrophobic interactions 100. The latest advance in this technology presents various detachable PEG-lipid conjugates (e.g., DSPE-PEG-maleimide, DSPE-PEG-folate, DSPE-PEG-biotin, DSPE-PEG-amine, and DSPE-PEG-carboxylic acid) for further conjugation purposes (antibodies/ligands, imaging molecules, therapeutic agents, and nanoparticles) 48. Furthermore, this lipid insertion method allows targeting ligands with a molecular weight larger than the lipid anchor to be efficiently conjugated on RBCm 47.

The second functionalization method is biotin-avidin bridges. This method is frequently used in surface engineering of cell membrane, mostly for indirect conjugation. The avidin-biotin complex is the strongest known non-covalent interaction, which is unaffected by extreme pH, temperature, organic solvents and other denaturing agents 50. The conjugation process (biotin-avidin) can be done at room temperature for an hour, and the unreacted sites of avidin/streptavidin can be inactivated by incubation with free biotin molecules and washed with PBS 52. However, because there are three major steps, including (1) biotinylation of RBCs and molecules of interest, (2) reaction with avidin/streptavidin, and (3) reaction with biotinylated molecules, so this method requires several times of RBC washing, leading to time consuming. Next, EDC/NHS coupling can be used to indirectly conjugate inorganic nanoparticles on RBCm. The disadvantage of direct chemical conjugation to RBCm is either time consuming or damaging RBCm, although it offers strong anchoring of the conjugated molecules. Two different research groups reported that UCNPs and MNPs could successfully be functionalized on the RBCm 64, 65. Interestingly, an antibody/ligand-receptor conjugation technique can be used not only for *ex vivo* functionalization, but also *in vivo* loading of therapeutic agents to circulating RBCs. RBCm proteins can be used as targeted receptors for ADCs (e.g., band 3, GPA, and CR-1) 66. The *ex vivo* loading is simple and just requires incubation for several hours to let the ADCs bind to the targeted receptors on RBCm. However, this conjugation technique is not strong compared to chemical conjugation. In addition, the use of scFv of monoclonal antibodies is preferred to target the RBC receptors as it does not cause the harmful immune responses compared with the murine monoclonal antibodies. This technique has recently been used to conjugate nanoparticles to RBCs with no adverse effects on the oxygenation status of RBCs 68. Currently, Xie et al. developed a technique to confirm the right-side-out orientation of RBCm coating by attaching a hidden peptide ligand derived from the cytoplasmic protein P4.2 (P4.2 peptide) to the band 3 proteins in RBCm. This technique is based on the specific interaction between the intracellular domain of band 3 transmembrane receptors in the inner side of RBCm and the P4.2 peptide ligand-modified nanoparticles 101.

The last discussed functionalization technique is passive adsorption (hitchhiking), commonly referred to as RBC-hitchhiking, which facilitate nanoparticle delivery through the transportation on RBCm via either hydrogen bonds, electrostatic interactions, van der walls forces, or hydrophobic interactions. Although the binding strength of this technique is much weaker when compared to covalent conjugation, this technique can be used to design smart drug delivery system with activation stimuli of high shear stress and cell/wall friction.

In the application of cancer treatments, engineered RBCs and RBCm-cloaked nanoparticles have recently demonstrated their significant outcomes in preclinical studies. In cancer chemotherapy, for instance, certain types of drugs (DOX, PTX, and GA) have been successfully encapsulated into RBCm-cloaked PLGA NPs and RBCm-cloaked CMC NPs. To improve its targeting, the surface of RBCm-cloaked nanoparticles could be functionalized with ligands/antibodies, such as anti-EGFR-iRGD recombinant protein 74, 80, 81. In cancer immunotherapy, some scientists have successfully developed nanovaccines to target delivery antigens to APCs. Such antigenic hgp100_25-33_ peptide and MPLA could be encapsulated into RBCm-cloaked PLGA NPs, whereas its surface could be functionalized with pMHC-1 and CD28 antibodies via a biotin-avidin bridge and active targeted mannose and IL2 via a lipid insertion method 52, 84. In cancer photodynamic/thermal therapy, EDC/NHS reaction could be used to functionalize UCNPs with RB-HA and RGD peptides, while biotin-avidin bridges facilitated the functionalized UCNPs to anchor to ICG-loaded RBCs for PDT 64. Interestingly, cell membrane coating technology allows us to fuse two or more types of cells for enhancing cancer targeting. For instance, RBCm could be fused with MCF-7 membranes for coating melanin nanoparticles for PTT 87.^73^

Owing to the significant advances in surface engineering of RBCm, now it is possible to not only functionalize RBCs and RBCm-cloaked nanoparticles for targeting cancer and imaging, but also to do at the same time theranostic nanomedicine with combined therapy to combat cancer. A number of studies have reported the combination of PDT/PTT with chemotherapy based on engineered RBCs and RBCm-cloaked nanoparticles 91-94. Furthermore, PTT can be combined with immunotherapy. This strategy can be used to induce cancer cell apoptosis via PTT and mobilize the immune system to eliminate the residual and metastatic cancer cells 95.

Instead of the use of RBC carriers and RBCm-cloaked nanoparticles, scientists also developed RBCm for direct encapsulation of drugs, known as RBCm-derived nanovesicles. Some techniques have already been developed to improve its drug loading efficiency, such as using cholesterol enrichment. However, reports on its surface functionalization, such as using lipid insertion and biotin/avidin methods, are still limited 102. Moreover, the use of exosomes for drug delivery is now on the rise of investigation. Reticulocytes, immature RBCs, are one of the major sources of exosomes in the bloodstream. However, there are still few reports on exosomes' functionalization for enhanced drug delivery to cancer. Recently, a newly developed technique has been proposed to simply isolate the RBC-derived exosomes and functionalize with magnetic nanoparticles to enhance the targeting ability. There are four major steps of functionalization, including (1) serum extraction, (2) exosome separation using transferrin-bound magnetic nanoparticles, (3) redispersion in PBS, and (4) drug loading 103.

In conclusion, the recent developments of surface functionalization in erythrocyte-based nanomedicine have demonstrated their superior properties in targeting, imaging, delivering drugs, and combining multiple treatments to synergistically combat cancer, thereby promoting the potential transition of this erythrocyte-based nanomedicine from bench to bedside as well as for the future of personalized cancer medicine.
